# Supplementary material for: Mutational Analysis of c-KIT and PDGFRA in Canine Gastrointestinal Stromal Tumors (GISTs)
Source: Vet Sci. 2022 Jul 21;9(7):376. doi: 10.3390/vetsci9070376 (PMC9323380; doi:10.3390/vetsci9070376)
Supplement: Supplementary file 1 [file vetsci-09-00376-s001.zip › vetsci-1819188-supplementary.pdf]

Supplementary Material: PCR methodological procedures

Analysis of c-kit and PDGFRA mutations

1. Genescanning (Fragment Analysis). The PCR products obtained with fluorophores labeled primers were diluted 1:20 with HPLC quality water (Fluka) Then 1 uL of diluted PCR was mixed by pipetting with the volumetric pipette with 0.1 uL of Genescan ® 500 LIZ Size Standard (Applied Biosystems) and 20 uL of Hi-di Formamide ® (Applied Biosystems). The mixture was then denatured at 95 °C for 5 'and the denatured PCR product was loaded onto the automatic sequencer with injection at 15Kv for 5 seconds. The sequencer capillary was previously loaded with POP-4 polymer (Applied Biosystems). The electrophoretic run took place at 60 °C using the electrophoresis module (Run Module): GS STR POP4 (1ml) G5.md5. The ride data was then analyzed using Genemapper ID vers3.2 (Applied Biosystems) or Peak Scanner v1.1 (Applied Biosystems) software.

2. DHPLC. To favor the formation of heteroduplexes, the amplified fragments were denatured at 95 °C for 10 minutes and then left to re-natural at room temperature for 15 minutes. The fragments were loaded onto the instrumentation for DHPLC (Wave DHPLC system, ADS Biotec, Omaha, USA) equipped with a column with patented technology (DNASep, ADS Biotec, Omaha, USA). The elution is carried out with a mixture of Buffer A (0.1 mol / l triethylammonium acetate – TEAA) and Buffer B (0.1 mol / l TEAA, 25% acetonitrile). For the optimal separation of heteroduplexes, different temperatures were empirically tested on the basis of the simulations of the melting profiles produced and of the analytical conditions by the Wavemaker software (ADS Biotec, Omaha, USA). In the final assay, the temperatures reported in **Table 1** were used for each fragment. Data analysis was conducted using Navigator software (ADS Biotec, Omaha, USA).

3. Direct sequencing. PCR products were sequenced after purification of PCR products by NucleoSpin® Gel and PCR Clean-up (Macherey-Nagel). 10 μL of PCR products were mixed with 20 μL of Buffer NTI, then transferred to NucleoSpin®Gel and PCR Clean-up column mounted on 2 mL tubes. The columns were then centrifuged for 30 seconds at 12000 rpm in the Eppendorf Mini-Spin centrifuge and the filtered liquid was removed. The columns were then washed twice by adding 700 μL of Buffer NT3 and centrifuging at 12000 rpm for 30 seconds. Finally, the purified PCR product was eluted from the membrane using 15 μL of Buffer NE. The purified PCR product was used as a template to set up a reverse primer sequencing reaction by Big Dye Terminator v 1.1 (Applied Biosystems) using a reaction mix as reported in **Table 1**. The reaction mixture was subjected to cycle-sequencing on a thermal cycler with the following protocol: 96 °C for 1 minute of initial denaturation, then followed by 40 cycles consisting of an initial phase of denaturation at 96 °C for 1 minute then annealing to the same PCR temperature for each primer for 5 sec and finally 60 °C for 4 sec. The mixture thus composed was subjected to the following protocol; the annealing temperature was the optimal one identified for each pair of primers in the PCR setup phase. The sequencing product was then purified by NucleoSEQ Columns (MACHEREY-NAGEL) and subjected to electrophoretic run on ABI PRISM® 310 Genetic Analyzer using a 46 cm diameter 50μm capillary filled with POP-4 polymer (Applied Biosystems). The data obtained were analyzed using specific software: Sequencing Analysis version 5.2.0 (Applied Biosystems) and algorithm for calling the bases (Basecaller) KB; the analysis software was set with a threshold of 15% for calling the 2nd peak.
